# Mechanistic Causes of Reduced Cardiorespiratory Fitness in Type 2 Diabetes

**DOI:** 10.1210/jendso/bvaa063

**Published:** 2020-06-07

**Authors:** Layla A Abushamat, P Mason McClatchey, Rebecca L Scalzo, Irene Schauer, Amy G Huebschmann, Kristen J Nadeau, Zhenqi Liu, Judith G Regensteiner, Jane E B Reusch

**Affiliations:** 1 Department of Medicine, University of Colorado, Anschutz Medical Campus, Aurora, Colorado; 2 Sigilon Therapeutics, Cambridge, Massachusetts; 3 Rocky Mountain Regional VA, Aurora, Colorado; 4 Center for Women’s Health Research, University of Colorado, Anschutz Medical Campus, Aurora, Colorado; 5 Department of Pediatrics, University of Colorado, Anschutz Medical Campus, Aurora, Colorado; 6 Department of Medicine, University of Virginia, Charlottesville, Virginia

**Keywords:** cardiorespiratory fitness, type 2 diabetes, cardiovascular disease, microvascular, mitochondria, endothelium

## Abstract

Type 2 diabetes (T2D) has been rising in prevalence in the United States and worldwide over the past few decades and contributes to significant morbidity and premature mortality, primarily due to cardiovascular disease (CVD). Cardiorespiratory fitness (CRF) is a modifiable cardiovascular (CV) risk factor in the general population and in people with T2D. Young people and adults with T2D have reduced CRF when compared with their peers without T2D who are similarly active and of similar body mass index. Furthermore, the impairment in CRF conferred by T2D is greater in women than in men. Various factors may contribute to this abnormality in people with T2D, including insulin resistance and mitochondrial, vascular, and cardiac dysfunction. As proof of concept that understanding the mediators of impaired CRF in T2D can inform intervention, we previously demonstrated that an insulin sensitizer improved CRF in adults with T2D. This review focuses on how contributing factors influence CRF and why they may be compromised in T2D. Functional exercise capacity is a measure of interrelated systems biology; as such, the contribution of derangement in each of these factors to T2D-mediated impairment in CRF is complex and varied. Therefore, successful approaches to improve CRF in T2D should be multifaceted and individually designed. The current status of this research and future directions are outlined.

The prevalence of diabetes mellitus (DM) in the United States has been rising since 1990. In 2018, 34.2 million people, around 10.5% of the US population, had DM (11% of men, 9.5% of women) [1]. In 2016, it was estimated that 91.2% of adults with DM have type 2 diabetes (T2D) [2] and the prevalence of youth-onset T2D is also rising [1]. DM and its related health complications, including cardiovascular disease (CVD), chronic kidney disease (CKD), and congestive heart failure (CHF), are associated with significant morbidity and mortality. People with DM accounted for 7.8 million hospital discharges in 2016 with 1.7 million of these hospitalizations due to major CVD events [1]. Additionally, premature mortality from CVD is 2- to 6-fold greater in people with DM than for people without diabetes [3]. Lower levels of cardiorespiratory fitness (CRF), measured as maximal oxygen consumption (VO_2max_), are predictive of greater short-term mortality risk in adults and children with DM [4–6], as in the general population [7–9]. Lower CRF in people with DM has also been associated with future CVD events and as such is an important modifiable CV risk factor [10–12]. In a study by Seyoum et al., men and women with diabetes who developed CVD events within 5 years had lower baseline peak VO_2_ than those who did not have CVD events during this follow-up period [10].

Compared with healthy individuals without diabetes, CRF is lower in adults and children with T2D and in children with type 1 diabetes, even in the absence of clinically apparent CVD in either group. These findings were observed in individuals with similar habitual physical activity levels, age, pubertal stage (for youth), and body mass index (BMI) (Table 1) [13–19]. Additionally, the presence of T2D confers a greater CRF deficit in women than in men [15, 20]. Not only does lower CRF predict premature mortality, it may lead to barriers to exercise recommendations by raising the relative intensity of a given work rate [21]. Potential physiological mechanisms that may contribute to lower CRF in people with T2D include insulin resistance and mitochondrial, vascular, and cardiac dysfunction. This review will focus on how each of these factors may contribute to CRF impairment in T2D and conclude with an assessment of the current state of knowledge about sex differences in CRF in people with T2D.

**Table 1.** Mean cardiorespiratory fitness (CRF) before graded exercise training in women with and without type 2 diabetes (T2D).

	Lean participants (control)	Overweight participants (control)	T2D participants
VO_2max_ (mL/kg/min)	25.1 ±4.7	21.8 ±2.9	17.7 ±4.0^*a*^

Values are means ±SD.

^*a*^ *P* < .05 for difference between the T2D group and the other 2 groups.

*Adapted* *from Brandenburg SL, Reusch JE, Bauer TA, Jeffers BW, Hiatt WR, Regensteiner JG. Effects of exercise training on oxygen uptake kinetic responses in women with type 2 diabetes. Diabetes Care. 1999;22(10):1640–1646* [17].

## 1. Search Methods

Articles included in this narrative review were compiled from original research articles, societal guidelines, and reviews from peer-reviewed journals included in the PubMed database as of 9 April 2020. Search terms included “exercise capacity” OR “cardiorespiratory fitness” PLUS “diabetes AND either “endothelial,” “mitochondria,” “blood flow,” “fibrinolysis,” OR “NOS.” We additionally searched references within publications relevant to the topic.

## 2. Insulin Action and CRF

Insulin resistance, a defining feature of T2D, correlates with decreased CRF. For example, we demonstrated that insulin resistance measured by the hyperinsulinemic euglycemic clamp strongly and independently correlated with peak VO_2_ in adolescents with T2D [18, 19]. In a proof of concept study, we therefore tested the impact of the insulin sensitizer rosiglitazone on CRF in people with T2D. We observed a significant 7% increase in peak VO_2_ with rosiglitazone alone compared with a placebo (Table 2) [22]. This finding was corroborated by Kadoglou et al., suggesting that targeting insulin action can improve functional status in people with T2D [23, 24]. Insulin action is associated with cardiac and skeletal muscle function and metabolic flexibility with exercise [25]. In addition, our current work supports a relationship between insulin action and factors related to CRF: cardiac function and skeletal muscle microvascular perfusion [20, 26]. As a vasoactive hormone, insulin has been shown in both rodents and healthy human participants to increase skeletal and cardiac muscle perfusion [27–29]. This perfusion response is blunted in insulin-resistant states [30, 31]. Decreased microvascular blood flow has been shown during insulin infusions in both insulin-resistant animal models and human participants [32]. Moreover, recent studies indicate that decreased microvascular blood flow may limit muscle glucose uptake by limiting the delivery of glucose to the myocyte [33]. Glucose and oxygen are both perfusion limited in their delivery to muscle, suggesting that any underlying microvascular defects are likely to have similar effects on both oxygen and glucose [34]. Even in people with obesity and no family history of DM, insulin-mediated microvascular perfusion in heart and skeletal muscle is decreased [35]. This is important as the microvasculature provides endothelial surface area needed for tissue uptake of oxygen and substrates that are vital to tissue health and function [36]. Indeed, multiple studies have shown that improved skeletal muscle microvascular perfusion via insulin sensitizers/enhancers and agents that cause vasodilation, such as glucagon-like peptide 1 receptor agonists (GLP-1), is associated with improved muscle oxygenation regardless of insulin sensitivity, suggesting that targeting microvascular insulin resistance and overall skeletal muscle and heart perfusion may result in both improved muscle oxygenation and glucose delivery during exercise [22, 37–39]. Taken together, these data support a role for insulin action in the heart, skeletal muscle, and the microvasculature as contributors to CRF.

**Table 2.** Mean cardiorespiratory fitness (CRF) before and after exercise when treated with thiazolidinedione (Rosiglitazone) vs placebo.

	Placebo	Rosiglitazone
VO_2max_ (mL/kg/min)		
Before	19.4 ± 5.2	19.8 ± 5.3
After	18.1 ± 5.3	21.2 ± 5.1^*a*^

Values are means ±SD.

^*a*^ *P* < 0.05 difference within groups before and after treatment.

*Adapted from Regensteiner JG, Bauer TA, Reusch JE. Rosiglitazone improves exercise capacity in individuals with type 2 diabetes. Diabetes care. 2005;28(12):2877–2883* [22]. *Copyright 2005 by the American Diabetes Association.*

## 3. Insulin Resistance and Oxidative Capacity

Insulin resistance is correlated with mitochondrial dysfunction, but the cause and effect relationship is unclear—whether this is a cause or consequence of insulin resistance is a matter of active investigation. For example, skeletal muscle oxidative enzymes are lower in adults with T2D, while glycolytic enzymes are elevated, compared with adults without T2D, suggesting a relationship between mitochondrial dysregulation and insulin resistance [40]. Insulin-resistant adult offspring of people with T2D also have lower mitochondrial activity in vivo and lower expression of mitochondrial and mitochondrial biogenesis genes and proteins than insulin-sensitive adults matched for physical activity, height, weight, and age [41–44]. In a study on healthy participants, a lipid infusion resulted in lower glucose oxidation and muscle glycogen synthesis, as well as a lower glucose-6-phosphate and intracellular glucose concentration, suggesting less glucose transport [45]. Evidence also demonstrates that lipid infusion induces insulin resistance by altering skeletal muscle microvascular perfusion [28]. Lower oxidative capacity has been previously shown in skeletal muscle with decreased NADH:O_2_ oxidoreductase activity (measure of respiratory chain activity, when normalized to citrate synthase and creatine kinase activity) in adults with T2D compared with both obese and lean adults [46]. Lower oxidative capacity has also been demonstrated in adolescents with T2D in vivo using in-MRI exercise. In particular, skeletal muscle adenosine diphosphate time constant, a blood flow–dependent mitochondrial function measure, was slowed and oxidative phosphorylation rates lower in the adolescents with T2D, linking skeletal muscle blood flow and mitochondrial dysfunction in these young people. Moreover, lack of suppression free fatty acids and longer skeletal muscle adenosine diphosphate time constant were independently associated with insulin resistance [47]. Therefore, the interplay between insulin resistance in skeletal muscle and mitochondrial dysfunction may be an adaptation to a chronically hyperglycemic and/or hyperlipemic state and is likely mediated by both microvascular and skeletal muscle changes [25, 48].

Mitochondrial dysfunction is also observed in the myocardium of people with T2D [49]. In human adults with well-controlled, uncomplicated T2D, there is a lower ratio of cardiac phosphocreatine to adenosine triphosphate than in healthy controls. Furthermore, young people and adults with T2D have lower indices of diastolic function, possibly suggestive of early diastolic dysfunction [18, 50]. These findings are similar to those seen in people with clinical heart failure [49, 51, 52]. Peterson et al. found that participants with T2D had lower fractional glucose uptake and oxidation, glycolysis, and glycogen deposition in myocardial tissue [53]. Impaired myocardial mitochondrial function in DM may correlate with future mortality. For instance, in people with dilated cardiomyopathy, the myocardial phosphocreatine-to-adenosine triphosphate ratio has prognostic value predicting total and CV mortality [54]. Our understanding of the complex interaction between insulin resistance, cardiac mitochondrial dysfunction, and CRF is still evolving [55].

## 4. Glucose, Endothelium, and Vascular Regulation

**Figure 1.**
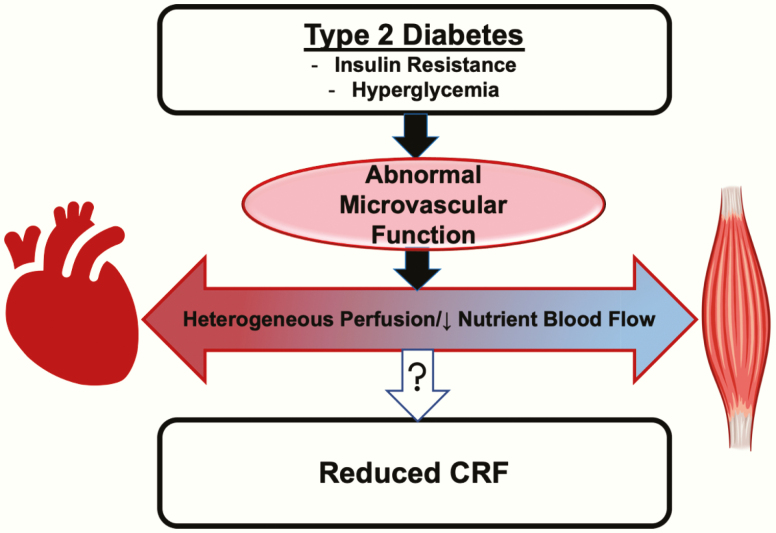
Insulin resistance and hyperglycemia of T2D lead to abnormal microvascular function and heterogeneous microvascular perfusion with lower nutrient blood flow, which may contribute to reduced CRF in people with T2D.

Hyperglycemia also leads to vascular dysfunction (Fig. 1). The endothelium, or lining of the vasculature, is the first defense of the tissue against toxic metabolites and inflammatory cytokines and is also responsible for moderating oxygen and nutrient access to tissue; both roles are imperative for tissue health and function [28, 56]. The endothelium fine tunes vascular tone via its production of nitric oxide (NO) [57], which leads to calcium-mediated vasodilation and increased perfusion of vital structures [57]. Insulin’s vasodilatory effect is NO mediated [58], and insulin increases NO production via Akt phosphorylation of endothelial nitric oxide synthase (eNOS) [59]. In T2D, insulin regulation of nitric oxide synthase (NOS) is impaired [59, 60].

In addition to insulin regulation of perfusion, DM impacts the macrovasculature and microvasculature in many ways. For example, elevation in glucose and fatty acids lead to inflammation and glucose-mediated production of advanced glycation end products (AGEs), activation of the renin–angiotensin activating system (RAAS) pathway, and aldosterone regulation [61]. Hyperglycemia leads to production of AGEs [62]. These accrue in the wall of vessels, leading to integral loss of the structure of the vessel wall and underlying basement membrane, as well as proinflammatory signaling contributing to systemic vascular dysfunction [63–65]. Additionally, in the Multiethnic Study of Arthrosclerosis (MESA) study population, higher aldosterone levels were associated with higher fasting glucose, insulin resistance, and risk of incident DM, suggesting a role for RAAS activation in DM [66]. Previous studies have shown that RAAS modulation with angiotensin II receptor blockers (ARBs) and angiotensin-converting enzyme inhibitors (ACEi) lower production of AGEs [67, 68]. Olmesartan, an ARB, has been shown to inhibit AGE-related inflammation in endothelial cells [69]. In the Heart Outcomes Prevention Evaluation (HOPE) study, treatment of participants with DM with ACEi led to lower risk of CVD events, mortality, and nephropathy [70]. Furthermore, Parving et al. showed the renoprotective effect of irbesartan, an ARB, on participants with DM and hypertension [71]. In the IRMA 2 study, inflammatory markers and endothelial dysfunction predicted progression to diabetic nephropathy in T2D [72]; participants on irbesartan, an ARB, had significantly lower markers of inflammation (high sensitivity c-reactive protein, interleukin-6, and fibrinogen) [73]. These findings suggest a role of AGEs and RAAS activation in vascular dysfunction and progression of microvascular disease in diabetes, which are likely contributors to impaired CRF as well.

Vascular dysfunction is present prior to overt CVD and is even detectable in youth with T2D. Decreased limb blood flow correlated with reduced peak VO_2_ in young people with T2D [18]. Additionally, arterial stiffness was seen in around half of the Treatment Options for Type 2 Diabetes in Adolescents and Youth (TODAY) cohort [74]. These data support a conceptual model wherein vascular insulin resistance, impaired eNOS regulation, and glucotoxicity- and lipotoxicity-mediated dysregulation of AGEs and vasoactive hormones could each contribute to decreased CRF in T2D.

## 5. The Role of NOS and NO in DM

Previous studies in healthy participants have shown that aerobic exercise attenuates mitochondrial damage and improves both vascular and skeletal muscle mitochondrial function, both of which are be impaired in DM [75–77]. However, when rat models of DM and hypertension (Spontaneously Hypertensive Heart Failure [SHHF] obese) underwent an exercise intervention, there was no induction of vascular mitochondrial content as expected and as seen with control rats [78]. NOS/NO are known upstream regulators of mitochondrial biogenesis [79–81]. Animal models have demonstrated that NOS/NO are pivotal to an expression of vascular mitochondrial content and adaptive response to exercise (Table 3) [38, 39, 78, 82–86]. Lack of NOS expression in these animal studies led to decreased vascular mitochondrial content and impaired vascular mitochondrial response to exercise [84] Therefore, eNOS has a role in mitochondrial adaptation to exercise, and dysregulation of eNOS in DM may explain the decreased response to exercise.

**Table 3.** Summary of animal studies investigating role of nitric oxide synthase (NOS) and nitric oxide (NO) in vascular mitochondrial adaptation to exercise, muscle perfusion and oxygenation, and muscle glucose extraction.

		Vascular mitochondria			Systemic response	
Animal study	Intervention	Content	Respiration	Adaptation	Endurance	Metabolism
Goto-Kakizaki (GK) rat^*a*^ [78]	High glucose	↓	↓	none	±↑	—
eNOS +/– and –/– null mice [84]	None	↓	—	—	—	—
Sprague Dawley rat^*b*^ [84]	NOS-inhibitor/exercise	↓	—	↓	—	—
GK rat^*a*^ [83]	Saxagliptin/exercise	↑	—	↑	↑	↑
Wistar rat^*c*^ [85]	GLP-1 receptor antagonist/exercise	—	↓	↓	↓	↓
Wistar rat^*c*^ [87]	BH_4_ precursor	↑	—	↑	—	↑
		**Skeletal muscle**				
		**Mitochondria**		**Perfusion/Oxygenation**		
Sprague Dawley rat^*b*^ [39]	High-fat diet/Liraglutide	↑		↑		
Sprague Dawley rat^*b*^ [38]	Continuous GLP-1 infusion	↑		↑		
Streptozotocin (STZ)-induced mouse^*a*^ [82]	BH_4_	↑		—		
eNOS and nNOS null mouse [86]	Acute and chronic exercise	↑±training response		—		

^*a*^Nonobese DM model.

^*b*^Metabolic syndrome model.

^*c*^Control model.

GLP-1 has been shown to stimulate NOS and cyclic AMP via G-protein–coupled receptor signaling, leading to increased glucose uptake in tissues and insulin-independent vasodilation [88]. In insulin-resistant rat models, treatment with liraglutide, a GLP-1 receptor agonist, rescued insulin-mediated increases in muscle perfusion and oxygenation [39]. Continuous GLP-1 infusion led to increased muscle microvascular blood perfusion, plasma NO, muscle insulin uptake, and muscle glucose extraction [38]. Furthermore, treatment with saxagliptin, a dipeptidyl peptidase 4 inhibitor that inhibits GLP-1 degradation, plus exercise training in an insulin-resistant rat model restored exercise-mediated vascular mitochondrial response and led to increased exercise capacity as measured by endurance [83]. Finally, rats treated with a GLP-1 receptor antagonist had decreased CRF, with and without exercise training. They also had attenuated vascular adaptation to exercise training [85]. However, in people with T2D, sitagliptin improved diastolic cardiac function, but not CRF, in the absence of formal exercise training [89]. Similarly, in people with T2D, treatment with exenatide, a GLP-1 agonist, improved diastolic heart function and arterial stiffness, but did not improve CRF in the absence of formal exercise training [90]. These data suggest a role for GLP-1 in improving insulin-mediated defects in mitochondrial function and endothelial function, as well as in mediating adaptive effects of exercise training; to date, these benefits have only been clearly demonstrated in animal models (summarized in Table 3).

It has been reported that eNOS, as 1 of 3 NOS isoforms, generates NO only if a key cofactor, tetrahydrobiopterin (BH_4_) is present [65]. NO originates from L-arginine catabolism by NOS. Reports have demonstrated that intravenous infusion of low-dose L-arginine improved insulin-mediated vasodilation in obese and T2D human participants and improved insulin sensitivity in all participants (healthy, obese, and T2D) [91]. Furthermore, our group treated participants with uncomplicated T2D with oral L-arginine for 7 days and demonstrated an increase in brachial artery diameter response and increased hyperemic forearm blood flow. There was no significant change in response noted in controls after 7 days of L-arginine [92]. In diabetes, oxidation of BH_4_ to 7,8-dihydrobiopterin leads to eNOS uncoupling and dysfunction [93]. In a metabolic syndrome mouse model, BH_4_ administration lowered glucose by acting as an insulin sensitizer and attenuating eNOS dysfunction [82]. Therefore, both defective insulin signaling to NOS and NOS uncoupling in diabetes can lead to abnormal vasomotion and vascular insulin resistance.

While NOS certainly has a role in vascular adaptation to exercise, it is not fully responsible for the lack of adaptive response to exercise training in animal models. eNOS and nNOS null mice subjected to acute (60 minutes) and chronic (9 days of 60 minutes) exercise training had increases in mitochondrial biogenesis markers during both short-term and long-term exercise training, demonstrating that while eNOS and nNOS are involved in mitochondrial adaptation, there are additional pathways necessary for mitochondrial response in the skeletal muscle to exercise training in nondiabetic models [86]. Sjøberg et al. have shown that these muscular mitochondrial adaptations to exercise are dependent on increased microvascular perfusion and molecular signaling, both induced by insulin, suggesting a role for blood flow–dependent insulin delivery and intact molecular signaling in the endothelium by insulin in exercise adaptive response and consequently decreased CRF in T2D [94].

## 6. Blood Flow and Muscle Oxygenation in T2D

Adequate perfusion of skeletal and cardiac muscle is necessary for appropriate function during a bout of exercise. Impaired blood flow distribution with exercise in T2D appears to contribute to decreased oxidative capacity by limiting skeletal muscle oxygen for oxidative flux. In a study by Bauer et al. [95], there was a rise in muscle deoxygenation (deoxygenated hemoglobin/myoglobin) that exceeded the rate of oxygen extraction after a bout of exercise in participants with T2D, suggesting a mismatch of supply to demand. There was also a delayed increase in blood flow after onset of exercise in these participants, consistent with perfusion limiting the exercise response [95]. Muscle deoxygenation during exercise in people with T2D was further studied by assessing maximal reactive hyperemic blood flow, peak oxygen utilization during exercise, and assessment of skeletal muscle oxygenation and its relation to blood volume and hematocrit. In control participants, muscle deoxyhemoglobin accumulation correlated inversely with peak VO_2_. In contrast, in people with uncomplicated T2D, this correlation was not present; we postulated that this difference in T2D was due to heterogeneous muscle blood flow distribution. In this study, participants with T2D had similar total limb blood flow and tissue hemoglobin content after a bout of exercise as those without T2D, suggesting that the oxygen extraction impairment is independent of limb total blood flow [26].

The role of heterogeneous microvascular perfusion in decreased skeletal muscle oxygen extraction in T2D was first tested in a metabolic syndrome animal model. We demonstrated that heterogeneous blood flow could predict oxygen extraction and that oxygen extraction could be restored with acute antioxidant treatment in obese Zucker rats [34, 96]. The observation that this defect could be corrected led to our next series of clinical studies. In sedentary obese participants with and without T2D, we examined in vivo oxidative flux using ^31^P-magnetic resonance spectroscopy. As with previous reports, we observed lower oxidative flux in people with T2D. We tested whether the difference in oxidative flux was due to lower oxygen availability using supplemental oxygen and observed that the supplemental oxygen improved oxidative flux in people with T2D, but not in control participants [97]. These data support the overall working model that perfusion heterogeneity and local muscle hypoxia contribute to in vivo impaired mitochondrial function in people with T2D.

## 7. Potential Mediators of Heterogeneous and Lower Nutrient Blood Flow in T2D

Heterogeneous blood flow in T2D may be in part due to degradation of the endothelial glycocalyx (Table 4), a semipermeable layer of glycoproteins and proteoglycans at the blood vessel luminal surface [104]. People with DM have been shown to have a decrease in glycocalyx in both acute and chronic exposure of their vessels to hyperglycemia. When there is loss of the glycocalyx, protective enzymes on the surface of the endothelium are lost, leaving endothelial cells vulnerable to oxidative stress and inflammation [98].

**Table 4.** Potential mediators of heterogeneous and lower nutrient blood flow.

T2D effects on blood flow
Endothelial glycocalyx degradation [98] Hypofibrinolysis [99, 100] Neutrophil extracellular trap formation [101] Capillary Rarefaction [96, 102, 103]

DM is also associated with premature atherosclerosis [105] and a prothrombotic state. Increased thrombosis is in part due to augmented platelet activation and creation of compact fibrin networks that are lysis resistant [99]. In our earlier work, we reported that premenopausal women with T2D lose their fibrinolytic potential [100]. Inadequate dynamic resolution of microthrombi is a plausible contributor to heterogeneous blood distribution. Additionally, people with DM have more neutrophil extracellular traps formation. Neutrophil extracellular traps are composed of histones and DNA, which interact with fibrinogen to form thicker fibrin fibers leading to prolongation of clot lysis. This association leads to augmentation of thrombin generation, reduction in permeability of the clot, and consequent decreased fibrinolysis. These complexes are correlated with glycemic control and increased inflammatory state (elevated interleukin-6) [101]. Taken together, these abnormalities are plausible contributors to heterogeneous blood flow in DM.

Lower capillary density, sometimes termed capillary rarefaction, is also present in DM. Reports by Prior et al. have demonstrated increased skeletal muscle capillarization with exercise training in elderly participants and people with impaired glucose tolerance [102, 103]. In a rodent model, we examined the relative contribution of capillary density versus heterogeneous tissue perfusion to muscle oxygen extraction. Only 20% of the decreased muscle oxygen extraction observed in the Zucker rat model could be explained by the lower skeletal muscle capillary density [96]. There is currently not a robust human literature on capillary density changes in uncomplicated T2D so more research is required.

## 8. Cardiac Dysfunction

We have reported abnormal cardiac function in the setting of exercise in people with uncomplicated T2D. Specifically, women with uncomplicated, recently diagnosed T2D have a significantly and disproportionately greater rise in pulmonary capillary wedge pressure during exercise than in healthy control female participants [20, 106]. This finding suggests a stiff heart, as is also seen with diastolic dysfunction. The augmentation of pulmonary capillary wedge pressure in response to exercise correlated with poorer myocardial perfusion across all regions of the heart [20]. Similarly, adolescents with T2D have greater left ventricular mass and abnormal cardiac circumferential strain, along with the finding of lower CRF than in lean and obese healthy control participants. Circumferential strain and CRF correlated with low adiponectin and fat mass, suggestive of obesity factoring into cardiac dysfunction and lower exercise capacity [19]. These findings of subclinical cardiac dysfunction with preserved cardiac output suggest that the noted decline in CRF in people with T2D may be independent of cardiac output and yet still have a cardiac contributor to its multifactorial etiology. Specifically, cardiac microvascular perfusion abnormalities contributing to cardiac stiffness may be implicated, but this question requires further evaluation [20].

## 9. Sex Differences in CVD Risk and CRF

There are similar absolute risks for CVD among men and women with DM. However, there is a much higher relative risk for CVD conferred by DM in women than in men with DM, despite a similar DM incidence [1, 107–110]. This difference in CV morbidity and mortality risk may be due to T2D dampening the cardioprotection thought to occur in premenopausal women [111–113]. It may also be due to a greater CVD risk factor burden in women with DM than in men with DM—conversely, it may also relate to a lower CVD risk factor burden in women without DM than in men without DM. The multifaceted underlying mechanisms that may account for the excess CVD risk seen in women with T2D are complex and are reviewed more comprehensively elsewhere [111, 114]. Furthermore, as described in the introduction, the presence of T2D confers a greater reduction in CRF in women than in men when compared with their nondiabetic counterparts [15, 20, 115]. Women of all ages have lower physical activity levels than their male counterparts [116, 117]. Women report more barriers to exercise, which may contribute to this difference in physical activity between the sexes [118, 119]. Women with T2D also perceive greater effort during exercise at the same work rate than women without T2D [21, 120]. To date, the physiological explanation for this sex difference is not well understood. Previous work demonstrated that female sex is associated with increased stiffness of the left ventricle during exercise among adults with T2D [121]. Additionally, in the HERITAGE study, women with T2D who underwent aerobic exercise training had a 3-fold smaller improvement in insulin sensitivity than men with T2D who completed the same intensity and frequency of exercise training [122]. It is unknown why these sex differences exist; further investigation is needed to assess underlying mechanisms that may contribute to lower CRF and higher relative risk of CVD in women with T2D.

## 10. Summary

DM prevalence continues to rise in the United States with associated increased CVD morbidity and mortality. People with T2D have reduced CRF compared with healthy participants, a factor that is associated with increased CV mortality. The mechanisms of lower CRF in T2D are multifaceted and involve interrelated defects in insulin action, mitochondrial dysfunction, skeletal muscle microvasculature, and cardiac dysfunction. Various interventions have been studied to counteract these abnormalities; however, further human research is needed to evaluate the effect of these therapies on CRF in T2D (Table 5). Additionally, there is variable response to intervention [123], perhaps due to the multifaceted complexity of exercise training and its effect on the heart, vasculature, and skeletal muscle. Regular exercise typically improves CRF and insulin resistance in healthy controls. However, exercise can have inconsistent therapeutic effect in people with T2D compared with otherwise similar healthy participants. Additionally, there appears to be a greater deficit in CRF and associated physiological dysfunction in women with DM. These sex differences suggest that more research should be done on the role of sex as a moderator of the response to exercise therapy. Therefore, interventions must focus on tackling these mechanisms contributing to reduced CRF, as well as augmenting the beneficial effects of exercise in people with T2D.

**Table 5.** Factors contributing to reduced cardiorespiratory fitness (CRF) in type 2 diabetes (T2D) and therapies that may improve these factors.

Factors	Potential interventions
Abnormal insulin action Lower oxidative capacity Impaired vascular regulation by nitric oxide synthase and nitric oxide Lower nutrient blood flow Heterogeneous microvascular perfusion Cardiac dysfunction Sex	Exercise [17, 123–125] Thiazolidinedione (rosiglitazone) [22, 23] Angiotensin II receptor blockers [67–69, 71, 73] Glucagon-like peptide 1 receptor agonists [36, 38, 39, 85, 88]Dipeptidyl peptidase 4 inhibitor [83]L-arginine (intravenous [91] or oral [92])

## Abbreviations

ACEi: angiotensin-converting enzyme inhibitor
AGE: advanced glycation end product
ARB: angiotensin II receptor blocker
BH4: tetrahydrobiopterin
BMI: body mass index
CHF: congestive heart failure
CKD: chronic kidney disease
CV: cardiovascular
CVD: cardiovascular disease
CRF: cardiorespiratory fitness
DM: diabetes mellitus
eNOS: endothelial nitric oxide synthase
GLP-1: glucagon-like peptide 1 receptor agonists
NO: nitric oxide
NOS: nitric oxide synthase
RAAS: renin–angiotensin activating system
T2D: type 2 diabetes
VO2max: maximal oxygen consumption

## Additional Information

***Disclosure Summary:*** J.E.B.R. and J.G.R. disclose AstraZeneca IIT and Merck IIT.

***Data Availability:*** Data sharing is not applicable to this article as no datasets were generated or analyzed during the current study.
